# Potent inhibitors of equine steroid isomerase EcaGST A3-3

**DOI:** 10.1371/journal.pone.0214160

**Published:** 2019-03-21

**Authors:** Helena Lindström, Aslam M. A. Mazari, Yaman Musdal, Bengt Mannervik

**Affiliations:** Department of Biochemistry and Biophysics, Arrhenius Laboratories, Stockholm University, Stockholm, Sweden; Weizmann Institute of Science, ISRAEL

## Abstract

Equine glutathione transferase A3-3 (EcaGST A3-3) belongs to the superfamily of detoxication enzymes found in all higher organisms. However, it is also the most efficient steroid double-bond isomerase known in mammals. *Equus ferus caballus* shares the steroidogenic pathway with *Homo sapiens*, which makes the horse a suitable animal model for investigations of human steroidogenesis. Inhibition of the enzyme has potential for treatment of steroid-hormone-dependent disorders. Screening of a library of FDA-approved drugs identified 16 out of 1040 compounds, which at 10 μM concentration afforded at least 50% inhibition of EcaGST A3-3. The most potent inhibitors, anthralin, sennoside A, tannic acid, and ethacrynic acid, were characterized by IC_50_ values in the submicromolar range when assayed with the natural substrate Δ^5^-androstene-3,17-dione.

## Introduction

Steroid hormones are involved in a plethora of physiological processes in mammals, ranging from regulation of blood pressure to reproduction [1]. Progesterone is a sex steroid hormone contributing to embryogenesis and maintaining pregnancy, it also plays an important role as an intermediate in metabolic pathways to other endogenous steroids [2]. Testosterone primarily exerts androgenic and anabolic effects in males [3]. These steroid hormones are mainly produced in testis, ovary, adrenal gland and placenta. However, some steroid hormones and their derivatives are also active in the nervous system where they are implicated in a variety of diverse physiological and pathophysiological conditions such as cognition, aggression, reproductive behavior, ageing, Alzheimer’s disease, Parkinson’s disease, and brain injury [4–6]. Steroid hormones synthesized in the brain and the nervous system are called “neurosteroids”, even though their chemical structures are identical to those of the cognate compounds produced in other tissues.

**Fig 1:**
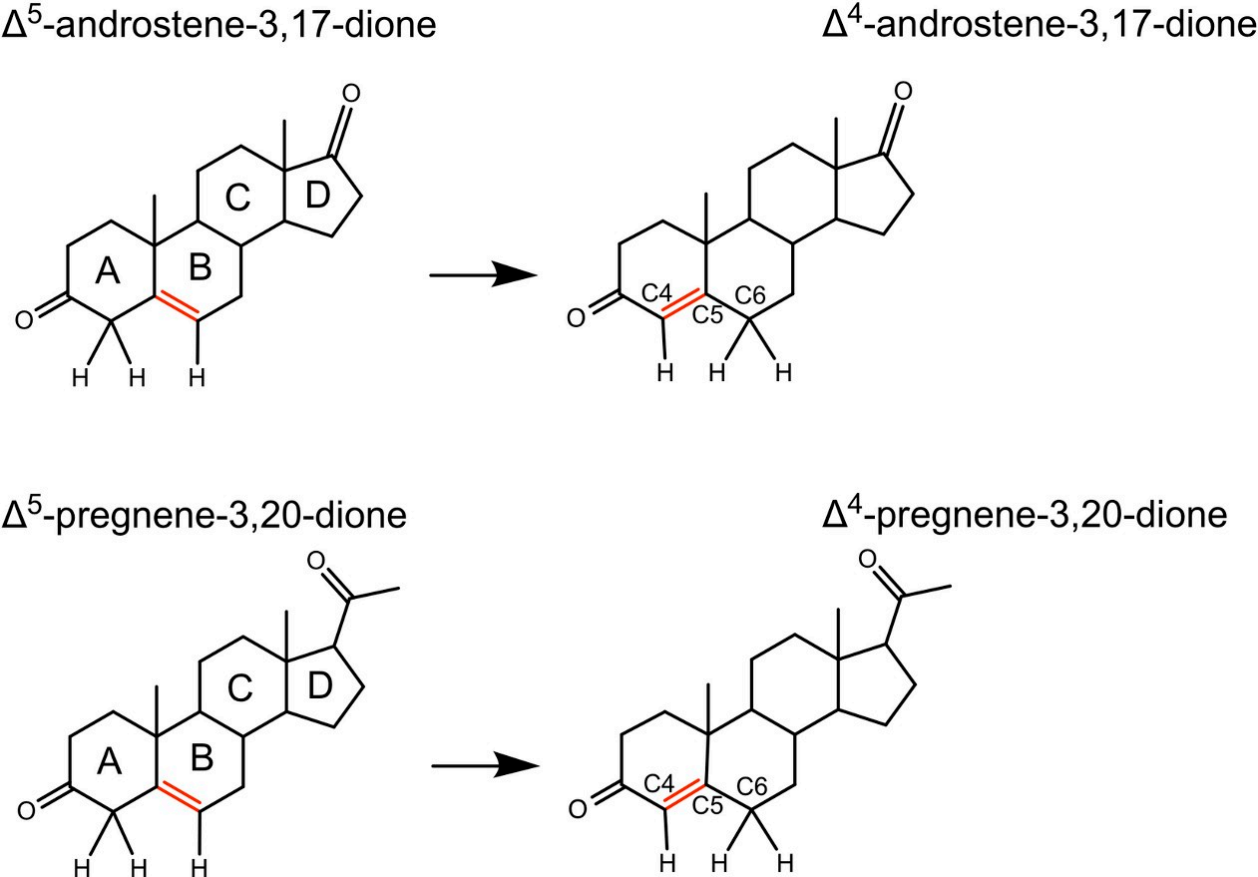
The steroid double-bond isomerization reactions catalyzed by GST A3-3 with the substrates Δ^5^-androstene-3,17-dione and Δ^5^-pregnene-3,20-dione.

The series of steroid biosynthesis reactions is catalyzed by a variety of enzymes including members of the cytochrome P450 superfamily as well as multiple isoforms of 3β-hydroxysteroid dehydrogenase (3βHSD) and 17β-hydroxysteroid dehydrogenase (17βHSD) [7]. Formation of Δ^4^-pregnene-3,20-dione (Δ^4^-PD, progesterone) and the proximate precursor of testosterone, Δ^4^-androstene-3,17-dione (Δ^4^-AD), are catalyzed by 3βHSD. This obligatory step includes an alcohol dehydrogenation followed by a Δ^5^- Δ^4^ double-bond isomerization. In *Homo sapiens* and *Equus ferus caballus*, the isomerization reaction has been shown to be even more efficiently catalyzed by another enzyme, GST A3-3, belonging to the alpha class of the glutathione transferase (GST, EC 2.5.1.18) superfamily (Fig 1). A porcine glutathione transferase has also been demonstrated to catalyze this reaction, albeit with lower efficiency [8–10].

Metabolites synthesized downstream of the GST-catalyzed isomerization are steroid hormones and neurosteroids with crucial functions in reproduction and in various aspects of wellbeing, and it is essential that they are kept at the adequate physiological concentrations. Overproduction of Δ^4^-AD and Δ^4^-PD under pathophysiological conditions could be suppressed by pharmacological intervention using GST inhibitors [11]. In the search for novel inhibitors we have screened a library of 1040 compounds, FDA-approved for various purposes.

Another important aspect of our investigation is the possible occurrence of unsuspected inhibitory side effects on steroid hormone production that may occur when the FDA-approved drugs are used for other pharmacological targets. The results of our present investigation could accordingly facilitate prediction of such adverse side effects.

## Materials and methods

### Materials

Δ^5^-AD was obtained from Steraloids Inc. (Newport, RI). The US Drug collection consisting of a set of 1040 FDA-approved compounds dissolved in DMSO was purchased from MicroSource Discovery Systems, Inc. (Gaylordsville, CT). All other chemicals were purchased from Sigma-Aldrich and Merck.

EcaGST A3-3 [10], HsaGST A3-3 [12] and HsaGST M2-2 [13] were cloned, expressed and purified as described previously. Protein concentration was determined by means of the Bradford assay [14].

### Enzyme inhibition assays

Δ^5^-AD was dissolved in methanol, CDNB in ethanol, and the compounds from the US Drug collection in DMSO. The final solvent concentration in the reaction system was kept at 5% (v/v) as a maximum.

The conjugation of GSH with the electrophilic substrate CDNB was used as a biochemical assay for general activity measurements. Screening of the US Drug library for inhibition was done in triplicates in 96-well plates in a Thermo Scientific Multiskan GO spectrophotometer. The conjugation activity at 30°C was monitored at 340 nm for 1 min in 0.1 M sodium phosphate buffer at pH 6.5 and 10 μM inhibitor concentration in a final volume of 300 μL. Initial concentrations of GSH and CDNB were 1 mM.

To obtain IC_50_ values of the most potent inhibitors identified in the screening, enzymatic activity with CDNB as well as with Δ^5^-androstene-3,17-dione (Δ^5^-AD) was monitored spectrophotometrically with a series of inhibitor concentrations. Measurements with Δ^5^-pregnene-3,20-dione (Δ^5^-PD) were not made due to solubility difficulties.

Assay conditions for inhibition measurements of enzyme activity with CDNB were the following: EcaGST A3-3 15 nM, initial concentrations of GSH and CDNB 1 mM, inhibitor concentrations varying from 0.029 μM to 15 μM, in 100 mM Na_2_HPO_4_/NaH_2_PO_4_ at pH 6.5. Assay conditions for inhibition measurements of enzyme activity with Δ^5^-AD were the following: EcaGST A3-3 0.8 nM, rather than the higher concentration used with CDNB, initial concentrations of GSH 1 mM and of Δ^5^-AD 0.1 mM, inhibitor concentrations varying from 0.0037 μM to 15 μM, in 25 mM Na_2_HPO_4_/NaH_2_PO_4_ at pH 8.0.

For determination of inhibition modality and K_i_ values of the most potent inhibitors, ethacrynic acid concentration was kept at 0.25 μM in the reaction with CDNB and anthralin concentration was kept at 0.09 μM in the reaction with Δ^5^-AD. GSH concentration was kept at 1 mM while the other substrate (CDNB or Δ^5^-AD) concentration was varied.

The reactions were followed spectrophotometrically for 1 min at 30°C using a Shimadzu UV-2501 PC spectrophotometer (Shimadzu Inc.). All experiments included triplicate measurements in each data point.

Due to the high activity of EcaGST A3-3 with steroid substrates the enzyme had to be diluted to nanomolar concentrations. With these low concentrations, adsorption of the enzyme to the inner walls of test tubes previously caused irreproducible measurement results [15]. Sarstedt micro-tubes 1.5 ml EASY-CAP (ref. no. 72.690.550) were found to give consistent results, and were subsequently used after numerous trials with tubes from various manufacturers.

### Data analysis

Molar absorption coefficients used for calculations were ε_248_ = 16.3 mM^-1^ cm^-1^ for the isomerization of Δ^5^-AD, and ε_340_ = 9.6 mM^-1^ cm^-1^ for the conjugation of CDNB.

IC_50_ values of the most potent inhibitors were obtained by plotting percentage remaining enzyme activity (y) versus log of at least seven inhibitor concentrations using the nonlinear regression option of the GraphPad Prism version 7 software. The default option of Hill slope = 1 was not chosen since many of the curves were better fit to the equation with a variable Hill slope:

$$y=y_{min}+\frac{y_{max}-y_{min}}{1+10^{\left(a-x\right)*n}}$$

where y_max_ and y_min_ denote the highest and the lowest activity, respectively, a = log(IC_50_), x = log [I], and *n* is the Hill coefficient.

The equation for competitive inhibition fitted by non-linear regression was

$$v=\frac{V_{max}\left[\text{S}\right]}{K_{M}\left(1+\frac{\left[\text{I}\right]}{K_{i}}\right)+\left[\text{S}\right]}$$

where [S] is the substrate concentrations and [I] is the inhibitor concentration.

Each data point is given as mean and standard deviation of triplicate measurements.

## Results

### Screening of the US drug library for inhibition of GST activity

**Fig 2:**
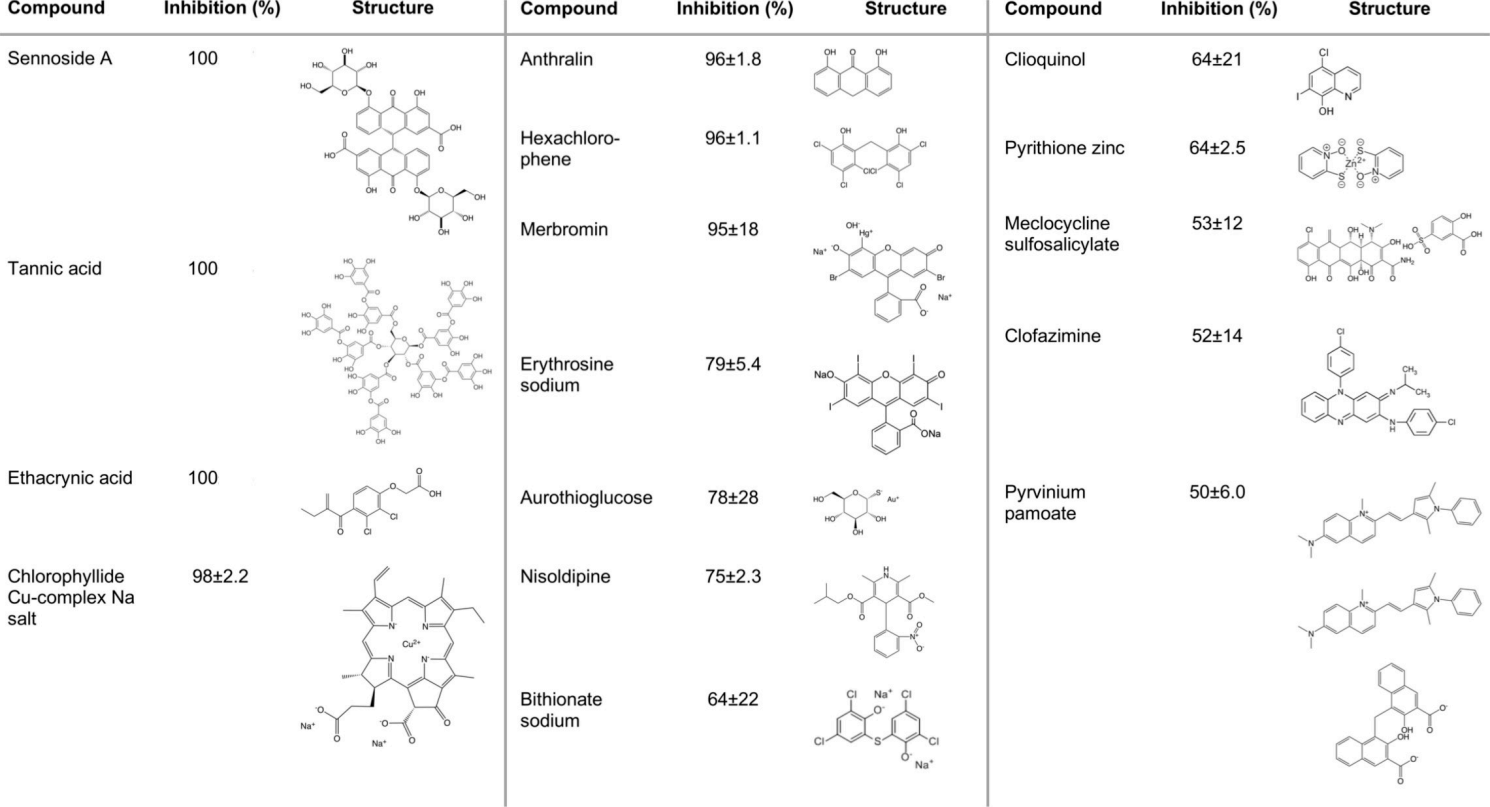
The most potent inhibitors (≥50% inhibition at 10 μM) from the US drug library screened with EcaGST A3-3. Enzymatic activity was measured with 1 mM GSH and 1mM CDNB with and without 10 μM inhibitor in sodium phosphate buffer at pH 6.5 and 30°C.

The chemical compound library was screened for inhibitory effects on EcaGST A3-3 activity using the universal GST substrate 1-chloro-2, 4-dinitrobenzene (CDNB). Screening at 10 μM inhibitor concentration revealed 16 compounds giving at least 50% inhibition (Fig 2) and 13 compounds giving inhibition between 30% and 50% (Table 1). Sennoside A, tannic acid, and ethacrynic acid were the strongest inhibitors yielding an inhibition of 100%.

**Table 1:** Compounds from the US drug library screened with EcaGST A3-3 giving 30% to 50% inhibition. Enzymatic activity was measured with 1 mM GSH and 1mM CDNB with and without 10 μM inhibitor in sodium phosphate buffer at pH 6.5 and 30°C.

Compound	Inhibition (%)
Sanguinarine sulfate	47±19
Metaproterenol	42±24
Exemestane	41±12
Chloramphenicol palmitate	40±1
Estropipate	37±23
Clarithromycin	36±7
Mebendazole	35±15
Nalbuphine hydrochloride	35±11
Lovastatin	33±15
Aminolevulinic acid hydrochloride	32±11
Alverine citrate	31±17
Fluvastatin sodium	31±24
Potassium p-aminobenzoate	30±16

### Determination of IC_50_ values with two alternative substrates

**Fig 3:**
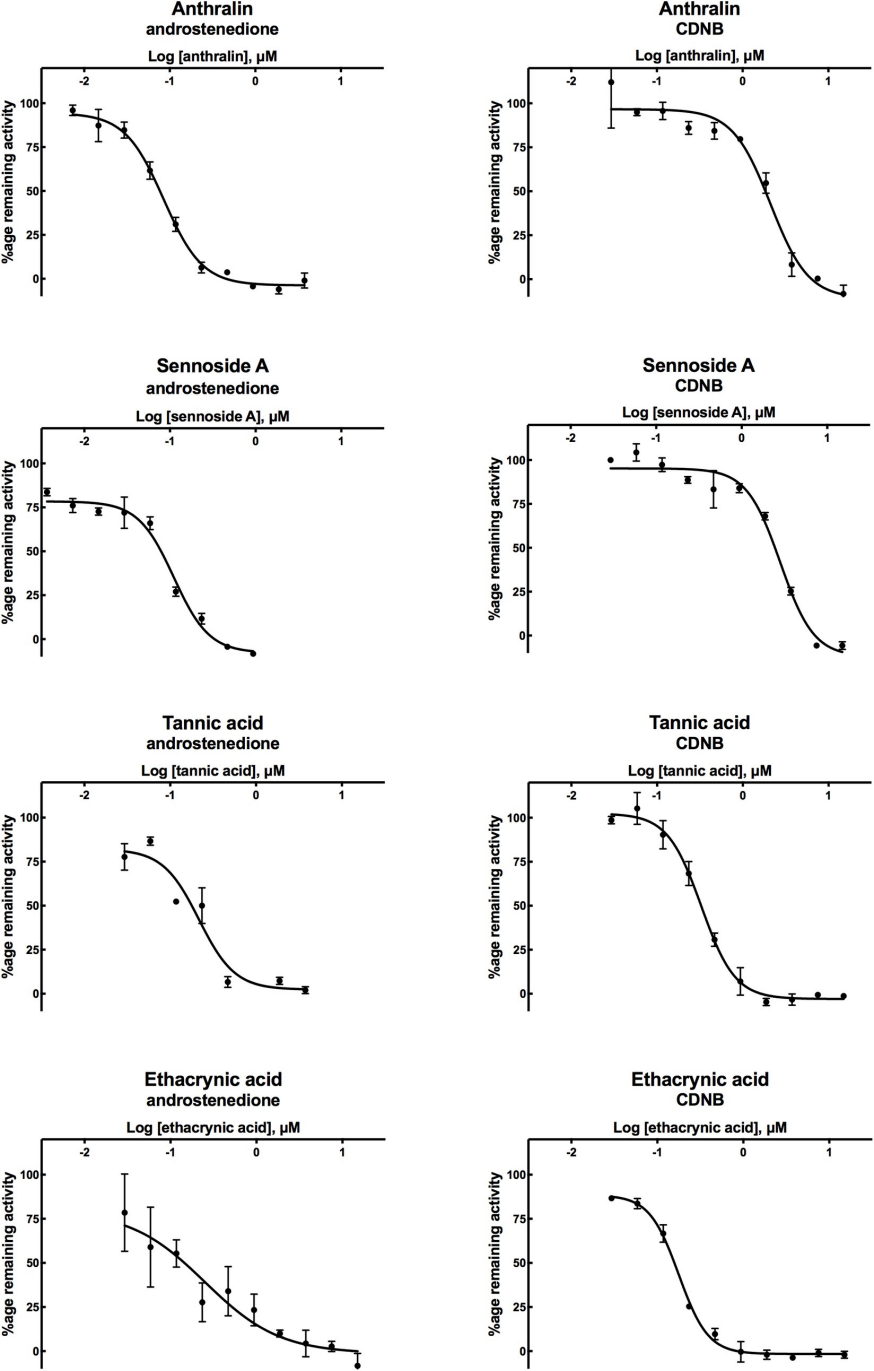
Dose-response curves of potent inhibitors of EcaGST A3-3 tested with the alternative substrates CDNB and Δ^5^-AD. Data points are means and standard deviations (SD) of triplicate measurements. The Hill coefficients represented by mean ± SD are for Δ^5^-AD inhibition: *n*_anthralin_ = 1.9±0.19, *n*_sennoside A_ = 2.1±0.29, *n*_tannic acid_ = 2.1±0.63, *n*_ethacrynic acid_ = 1.0, and for CDNB inhibition: *n*_anthralin_ = 2.0±0.43, *n*_sennoside A_ = 2.2±0.41, *n*_tannic acid_ = 2.2±0.23, *n*_ethacrynic acid_ = 2.5±0.21. The curve fitting for ethacrynic acid with androstenedione was not improved by using the Hill coefficient as a parameter and was therefore pre-set = 1.0 and presented no SD.

IC_50_ values of the eleven most potent inhibitors were determined using CDNB as substrate by varying the inhibitor concentration from 0.029 μM to 15 μM. In addition, IC_50_ values of these inhibitors with the natural substrate Δ^5^-AD were determined by varying the inhibitor concentration from 0.0037 μM to 15 μM (Fig 3 and Table 2). Ethacrynic acid, hexachlorophene and tannic acid were the most potent inhibitors of EcaGST A3-3 with CDNB, yielding IC_50_ values of 0.18, 0.32 and 0.33 μM, respectively. Enzymatic activity with Δ^5^-AD was most effectively inhibited by anthralin (IC_50_ = 0.085 μM), sennoside A (IC_50_ = 0.11 μM), and tannic acid (IC_50_ = 0.22 μM). In general, the inhibitory effects were similar irrespective of the substrate used in the assay. However, it is noteworthy that anthralin and sennoside A were both approximately 25 times more potent when used with Δ^5^-AD as substrate.

**Table 2:** IC_50_ values of the most potent inhibitors of EcaGST A3-3. Activities were tested with the alternative substrates CDNB (1 mM, in 100 mM sodium phosphate buffer) and Δ^5^-AD (0.1 mM, in 25 mM sodium phosphate buffer) in the presence of 1 mM GSH at 30°C. Inhibitor concentrations varied between 0.029 μM and 15 μM at pH 6.5 (for CDNB) and between 0.0037 μM and 15 μM at pH 8.0 (for Δ^5^-AD). The four most potent inhibitors with either substrate are highlighted. With some inhibitors high absorbance or other physical factors gave anomalous values.

	IC_50_ (μM)	
	CDNB	Δ^5^-AD
Ethacrynic acid	**0.18±0.067**	**0.25±0.10**
Hexachlorophene	**0.32±0.036**	0.44±0.22
Tannic acid	**0.33±0.018**	**0.22±0.034**
Aurothioglucose	**0.85±0.12**	Anomalous curve
Bithionate Na	1.9±0.52	1.6±0.31
Chlorophyllide Cu-complex Na salt	2.1±0.27	Anomalous curve
Anthralin	2.1±0.27	**0.085±0.0048**
Merbromin	2.6±0.63	2.5±0.48
Sennoside A	2.8±0.28	**0.11±0.0082**
Erythrosine Na	8.1±2.4	Anomalous curve
Nisoldipine	Anomalous curve	Anomalous curve

The highest IC_50_ value in Table 2 determined for EcaGST A3-3 with CDNB as substrate was that of erythrosine sodium (IC_50_ = 8.1 μM), and with Δ^5^-AD as substrate merbromin gave the highest value (IC_50_ = 2.5 μM).

Conspicuously, the majority of the inhibition curves for the most potent inhibitors were not hyperbolic with respect to inhibitor concentration, but showed Hill coefficients close to 2 (Fig 3). This apparent positive cooperativity was noted both with CDNB and Δ^5^-AD as substrate.

### Comparison of inhibitory effects on different GSTs

For understanding the effects of pharmacological interventions based on GST inhibitors, it is important to know the selectivity among the numerous GSTs. The percentage inhibition of the 11 most potent inhibitors of EcaGST A3-3 was compared to the corresponding values for HsaGST A3-3 and HsaGST M2-2, as well as for HsaGST P1-1 [12] and HsaGST S1-1 [16] investigated previously. The inhibitory effects on the equine and the human GST A3-3 were similar, such that they both differ from the effects of some inhibitors on human GSTs M2-2, P1-1, and S1-1 (Table 3). For example, 10 μM ethacrynic acid gave 94–100% inhibition of EcaGST A3-3 and HsaGST A3-3, but had low or negligible effects on GSTs P1-1, S1-1, and M2-2. All values were determined with CDNB, which is a common substrate for all the enzymes.

**Table 3:** Comparison of the effects of the most potent inhibitors of EcaGST A3-3 with their inhibitory effects on HsaGST A3-3, HsaGST M2-2, HsaGST P1-1, and HsaGST S1-1. The CDNB activities were measured with concentrations of the substrates GSH and CDNB at 1 mM, while inhibitor concentrations were 10 μM (HsaGST A3-3, HsaGST M2-2, and HsaGST S1-1) and 3.3 μM (HsaGST P1-1) in 0.1 M sodium phosphate buffer at pH 6.5 and 30°C.

	Inhibition (%)				
	EcaGST A3-3	HsaGST A3-3	HsaGST M2-2	HsaGST P1-1^a^	HsaGST S1-1 ^b^
Sennoside A	100	90	n. i.	< 4	30
Tannic acid	100	100	86	< 4	100
Ethacrynic acid	100	94	0	25	32
Chlorophyllide Cu-complex Na salt	98 ±2.2	84	100	51	100
Anthralin	96 ±1.8	88	80	22	84
Hexachlorophene	96 ±1.1	89	98	34	61
Merbromin	95 ± 18	98	85	44	85
Erythrosine Na	79 ±5.4	80	27	< 4	100
Aurothioglucose	78 ± 28	96	85	< 4	24
Nisoldipine	75 ±2.3	70	35	14	42
Bithionate Na	64 ± 22	47	73	10	47

^a^[12]

^b^[16]

n. i. = inhibition lower than 30%.

A more stringent analysis was based on IC_50_ values. Table 4 summarizes IC_50_ data determined with the most potent EcaGST A3-3 inhibitors and the values obtained for the other GSTs. In addition, the IC_50_ values of the most potent inhibitors of GSTs M2-2 and S1-1 are listed. The latter inhibitors have negligible effect on equine and human GST A3-3. The most potent inhibitors of HsaGST A3-3 with CDNB were hexachlorophene, tannic acid and chlorophyllide Cu-complex Na salt with the IC_50_ values 0.16 μM, 0.21 μM and 0.33 μM, respectively, values similar to those obtained for EcaGST A3-3.

**Table 4:** Comparison of IC_50_ values of the most potent inhibitors of EcaGST A3-3, HsaGST A3-3 and HsaGST M2-2 with published values for HsaGST P1-1 and HsaGST S1-1. Data are based on CDNB activities with GSH and CDNB concentrations constant at 1 mM in 0.1 M sodium phosphate buffer at pH 6.5 and 30°C.

	IC_50_ (μM)				
	EcaGST A3-3	HsaGST A3-3	HsaGST M2-2	HsaGST P1-1^a^	HsaGST S1-1^b^
Ethacrynic acid	0.18±0.067	0.4^a^	-	4.9	44
Hexachlorophene	0.32±0.036	0.16^a^	1.5	9.7	8.9
Tannic acid	0.33±0.018	0.21	1.2	-	0.35
Aurothioglucose	0.85±0.12	0.91	12	-	-
Bithionate Na	1.9±0.52	0.7^a^	-	-	12
Chlorophyllide Cu-complex Na salt	2.1±0.27	0.33^a^	-	2.3	1.7
Anthralin	2.1±0.27	0.72	-	-	24
Merbromin	2.6±0.63	0.66^a^	4.9	3.1	3.3
Sennoside A	2.8±0.28	0.98	-	-	39
Erythrosine Na	8.1±2.4	2.2	-	-	0.18
Nisoldipine	*	4.1	-	-	29
Sulfasalazine	-	-	0.30	-	-
Suramin	-	-	0.62	-	0.3
Hydroxyzine pamoate	-	-	0.76	-	-
Pyrvinium pamoate	-	-	1.2	-	17
Pyrantel pamoate	-	-	1.4	-	-
Oxantel pamoate	-	-	1.7	-	-
Benzoylpas	-	-	2.5	-	-
Pioglitazone	-	-	5.9	-	-
Sulindac	-	-	23	-	-

^a^[12]

^b^[16]

- = low inhibition in the initial screening of the library, no IC_50_ value determined.

* = anomalous curve.

The strongest inhibitors of the enzymatic activity of HsaGST M2-2 with CDNB were sulfasalazine (IC_50_ = 0.30 μM), suramin (IC_50_ = 0.62 μM), and hydroxyzine pamoate (IC_50_ = 0.76 μM). These compounds did not cause inhibition above 30% of EcaGST A3-3. The lowest IC_50_ values for HsaGST S1-1 were 0.18 μM and 0.3 μM for erythrocine sodium and suramin, respectively.

### Determination of inhibition modalities

**Fig 4:**
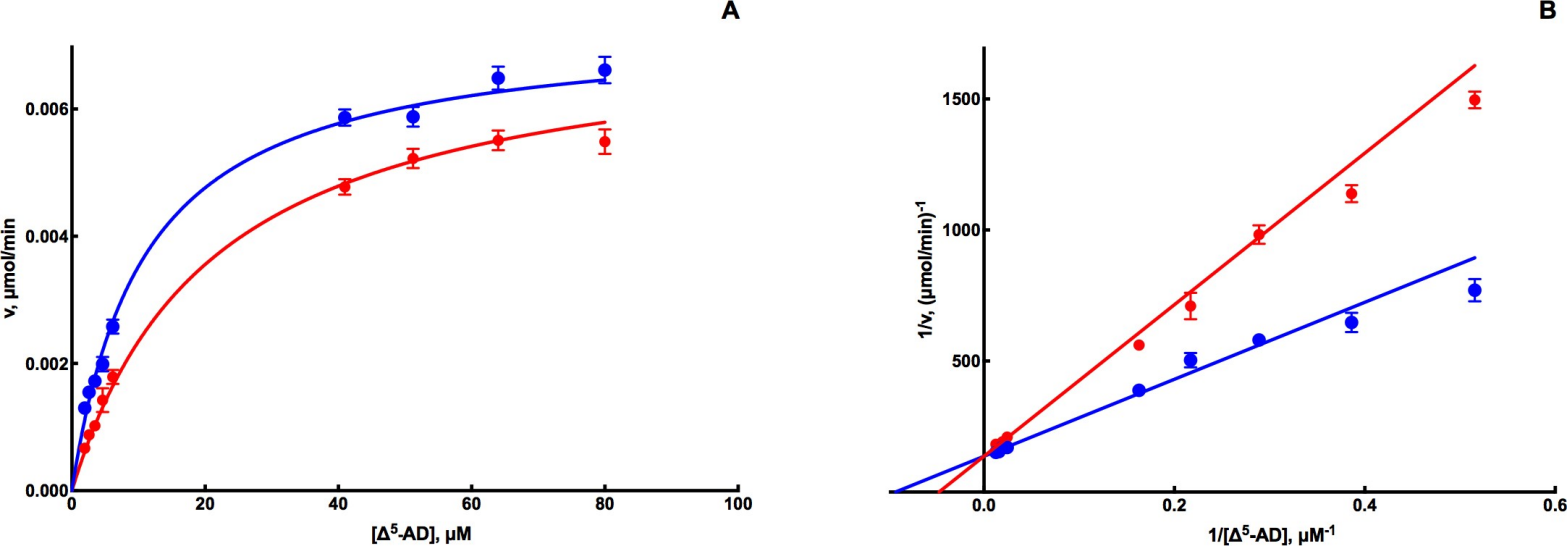
Substrate-saturation curves (A) and double reciprocal plot (B) of the most potent inhibitor anthralin with EcaGST A3-3 at varied concentrations of Δ^5^-AD. Data points are represented as means and standard deviations (SD) of triplicate measurements. The reaction was followed spectrophotometrically for 1 min at 1 mM GSH in the absence (blue) and presence (red) of 0.09 μM anthralin.

To evaluate inhibition modalities and K_i_ values of the two most potent inhibitors of EcaGST A3-3, the effect of ethacrynic acid and anthralin was measured at varying concentrations of the alternative substrates CDNB and Δ^5^-AD, respectively. Both inhibitors exhibited competitive behavior, illustrated by the convergence at the Y-axis in the double reciprocal plots (see Fig 4). The graphs are based on the Michaelis-Menten model in the version describing linear competitive inhibition, and the equation was fitted by nonlinear regression to the entire data set obtained with and without inhibitor. Ethacrynic acid yielded a K_i_ value of 0.14 μM and the K_i_ of anthralin was 0.09 μM (Table 5).

**Table 5:** Steady-state kinetic parameters of the most potent inhibitors of EcaGST A3-3.

Inhibitor	Substrate	K_*m*_ (μM)	K_*i*_ (μM)	Inhibition modality
Ethacrynic acid	CDNB	1260 ± 82	0.141 ± 0.0071	Competitive
Anthralin	Δ^5^-AD	10.8 ± 0.46	0.0930 ± 0.0078	Competitive

## Discussion

EcaGST A3-3 is the most efficient steroid double-bond isomerase known in mammals [10]. In the biosynthesis of steroid hormones, EcaGST A3-3 catalyzes the double-bond isomerization reaction from Δ^5^-PD to Δ^4^-PD as the last step in the synthesis of progesterone (i.e. Δ^4^-PD). Similarly, it catalyzes the transformation of Δ^5^-AD into Δ^4^-AD, where Δ^4^-AD is the ultimate precursor of testosterone. Among the main functions of progesterone is maintenance of pregnancy, whereas testosterone is the primary male sex hormone. In the nervous system, as in other tissues, other hormones are synthesized downstream of the steroid double-bond isomerization reaction catalyzed by EcaGST A3-3. Steroid hormones and their derivatives also play important roles as neurosteroids and are implicated in a number of physiological and pathophysiological processes [17,18]. For example, progesterone promotes myelination and dendritic growth, allopregnanolone increases hippocampal neurogenesis and estradiol regulates synaptic plasticity [19]. Thus, EcaGST A3-3 and the corresponding human HsaGST A3-3 could exert significant functions in the synthesis of neurosteroids and are potential pharmaceutical targets not only in reproductive disorders, but also in pathologies associated with the nervous system.

The US Drug collection is an assembly of 1040 diverse natural and synthetic FDA-approved compounds including chemotherapeutics, vasodilators, herbicides, diuretic, anti-inflammatory, antibacterial and antifungal agents. In the present study this set was investigated for inhibition of EcaGST A3-3. The rationale for this screening is that already approved compounds could proceed to clinical use on a faster route compared to *de novo* developed drug candidates. Conversely, the compounds already in use might exert undesired, not foreseen, side effects on EcaGST A3-3.

Among the tested compounds, 16 compounds inhibited EcaGST A3-3 reaction with the universal substrate CDNB by at least 50% (Fig 2). The 11 most potent inhibitors were subjected to further investigation for determination of the IC_50_ values with CDNB and the natural substrate Δ^5^-AD. In the reaction with CDNB, the four most potent of these compounds demonstrated IC_50_ values in the sub-micromolar range; six were in the low micromolar range and one generated an anomalous dose-response curve. With Δ^5^-AD, five were in the sub-micromolar range and two in low micromolar range, while four generated anomalous dose-response curves probably due to assay disturbances (Table 2). It should be noted that the strongest inhibitors were not equally potent with the alternative substrates CDNB and Δ^5^-AD.

The well-characterized diuretic ethacrynic acid [20] was identified as a strong inhibitor of EcaGST A3-3 with both CDNB and Δ^5^-AD, being the most potent of all the inhibitors with CDNB. Interactions of ethacrynic acid with GSTs have been investigated previously, and the compound has been found to be both a substrate to and an inhibitor of GSTs, inhibiting several human GSTs [12,16,21–24].

Tannic acid was another of the four most potent inhibitors of activities with both CDNB and Δ^5^-AD. Like ethacrynic acid, this compound has been identified as a GST inhibitor in previous studies. It has been suggested to have potential for use as an anticancer agent in treatment of cholangiocarcinoma [25] and to have a role as scavenger of active carcinogen metabolites, as well as to modulate the enzymes involved in the activation of xenobiotics and/or detoxication pathways [26].

The two strongest inhibitors with Δ^5^-AD were anthralin and sennoside A (Table 2). Anthralin is an antipsoriatic [27] and sennoside A is used as a cathartic [28]. These compounds have not been subjected to extensive studies involving GSTs and have not been detected as strong GST inhibitors previously.

The inhibition profiles obtained with the two alternative substrates exhibit similarities. Two of the four most potent inhibitors with either substrate are the same: ethacrynic acid and tannic acid. Interestingly, however, the two top inhibitors of Δ^5^-AD, anthralin and sennoside A, are found in the lower half of Table 2, where the inhibitors are listed in decreasing potency of CDNB inhibition.

The reason for the high affinity of sennoside A and anthralin can be sought in their structural similarities with the steroid substrates (Figs 1 and 2). Anthralin constitutes a part of sennoside A, and the anthracene portion of both compounds have a resemblance to Δ^5^-AD and Δ^5^-PD. This structural resemblance is found also in many other potent compounds (Fig 2). What distinguishes anthralin and sennoside A from the rest of the compounds is the presence of the adjacent ketone- and hydroxy-groups on the anthracene structure. Possibly, these functional groups are favorably positioned to interact with polar elements in the hydrophobic H-site of EcaGST A3-3. The low IC_50_ value of anthralin might also be partly due to entropic effects. The rigid molecule has no rotational energy, lowering the entropy loss upon formation of the EcaGST A3-3 –anthralin complex and contributing to a lower dissociation constant [29].

In general, it cannot be taken for granted that enzyme inhibitors are exerting their action by binding to the active site. Even competitive inhibition can be accomplished by allosteric binding, provided that the inhibitor induces a conformational change of the enzyme that prevents the binding of the substrate. Given the structural similarity of the inhibitors to the steroid substrate, however, the competitive binding likely involves the active site in the present investigation.

Dose-response curves of the most potent inhibitors of EcaGST A3-3 with Δ^5^-AD are shown in Fig 3, with the corresponding curves for the reaction with CDNB for comparison. A more thorough examination of the curves reveals Hill coefficients near 2 for all inhibitors shown except that of ethacrynic acid with Δ^5^-AD as substrate (Fig 3). One explanation for the corresponding steep curves in the semi-logarithmic plots could be cooperativity between the subunits of the homodimeric enzyme. Similarly, in the case of HsaGST P1-1 the presence of positive as well as negative cooperativity has been unveiled with certain substrates and inhibitors [30–34].

Originally, the subunits of soluble GSTs were found to be kinetically independent in studies based on inhibition of homo- and hetero-dimeric GSTs and measurements of their activity with various substrates [35,36]. Apparently, the experimental conditions, including the choice of substrates and inhibitors, determine whether cooperativity is displayed. A detailed investigation of the Alpha class GST A1-1, homologous to GST A3-3, demonstrated that the substrate used in the assay determined whether the enzyme displays half-of-the-sites or all-of-the-sites reactivity [37]. Apparently, the ligand bound to the enzyme governs the display of cooperativity. In inhibition studies of GST P1-1 involving ethacrynic acid unusual kinetic behavior was induced [33], and the effect of ethacrynic acid observed in the present study warrants further examination. However, this aspect is beyond the scope of this paper. Nevertheless, it should be emphasized that Hill coefficients were near 1.0 for the less potent inhibitors in the present investigation.

Inhibition data of EcaGST A3-3 and four human enzymes with CDNB are compared in Table 4. Many of the most potent inhibitors of EcaGST A3-3 are also potent with the other GSTs. Apart from this similarity is the striking difference of HsaGST M2-2 from the other enzymes. The most potent inhibitors of HsaGST M2-2 are not found among the strong inhibitors of other enzymes (with the exceptions of suramin and pyrvinium pamoate with HsaGST S1-1). A closer inspection of the molecular structures of the most potent HsaGST M2-2 inhibitors reveals that the structures do not resemble steroid hormones. Instead, they exhibit similarity to *o-*quinones, GST M2-2 substrates derived from dopamine and dopa [38,39], suggesting that the inhibitors act through competitive binding at the H-site, also in the case of HsaGST M2-2.

The inhibition profile of EcaGST A3-3 acquired from our measurements with its natural steroid substrates reveals that some of the substances in clinical use may exert side effects on equine steroid biosynthesis and thus might affect reproductive and other functions in the horse. Although measurements were not made with Δ^5^-PD in the current investigation, it should appear obvious that Δ^5^-PD double-bond isomerization is inhibited in an analogous manner as the Δ^5^-AD isomerization. Similarities between EcaGST A3-3 and HsaGST A3-3 inhibition profiles suggest that these side effects might also affect human steroid biosynthesis in a comparable manner. Because *Homo sapiens* and *Equus ferus caballus* share the Δ^5^-steroidogenic pathway for testosterone biosynthesis, whereas rodents use the Δ^4^-steroidogenic pathway [40], the horse appears to serve as a better animal model than rodents for investigations of therapeutic applications of GST A3-3 inhibitors.

## Conclusions

We have identified FDA-approved compounds already in clinical and other use as potent submicromolar-range inhibitors of EcaGST A3-3. Given the important role this enzyme appears to play in steroidogenesis, it can be a potential pharmaceutical target in treatment of disorders in the reproductive and nervous systems. Conversely, the identification of potent inhibitors may explain or predict possible undesired side effects on steroidogenesis exerted by the named compounds when they are administered for other purposes.
